# Pentraxin 3 plasma levels at graft-*versus*-host disease onset predict disease severity and response to therapy in children given haematopoietic stem cell transplantation

**DOI:** 10.18632/oncotarget.13488

**Published:** 2016-11-21

**Authors:** Erica Dander, Paola De Lorenzo, Barbara Bottazzi, Paola Quarello, Paola Vinci, Adriana Balduzzi, Francesca Masciocchi, Sonia Bonanomi, Claudia Cappuzzello, Giulia Prunotto, Fabio Pavan, Fabio Pasqualini, Marina Sironi, Ivan Cuccovillo, Roberto Leone, Giovanni Salvatori, Matteo Parma, Elisabetta Terruzzi, Fabio Pagni, Franco Locatelli, Alberto Mantovani, Franca Fagioli, Andrea Biondi, Cecilia Garlanda, Maria Grazia Valsecchi, Attilio Rovelli, Giovanna D'Amico

**Affiliations:** ^1^ “M. Tettamanti” Research Center, Pediatric Department, University of Milano-Bicocca, Monza, Italy; ^2^ Center of Biostatistics for Clinical Epidemiology, School of Medicine and Surgery, University of Milano-Bicocca, Monza, Italy; ^3^ Istituto di Ricovero e Cura a Carattere Scientifico (IRCCS) - Humanitas Clinical and Research Center, Rozzano, Italy; ^4^ Pediatric Onco-Haematology, City of Science and Health of Turin, Regina Margherita Children's Hospital, Torino, Italy; ^5^ Clinica Pediatrica Ospedale S. Gerardo, Fondazione MBBM, University of Milano-Bicocca, Monza, Italy; ^6^ Sigma-tau S.p.A., Department of R&D, Biotechnology, San Gerardo Hospital, Monza, Italy; ^7^ Haematology Division and BMT Unit, San Gerardo Hospital, Monza, Italy; ^8^ Department of Surgery and Interdisciplinary Medicine, University Milano-Bicocca, Section of Pathology, San Gerardo Hospital, Monza, Italy; ^9^ Department of Pediatric Haematology-Oncology, IRCCS, Bambino Gesù Children Hospital, Roma-Department of Pediatric Science, University of Pavia, Pavia, Italy; ^10^ Humanitas University, Rozzano, Italy

**Keywords:** acute graft-versus-host disease, pentraxin 3 (PTX3), biomarkers, pediatric haematopoietic stem cell transplantation, hematology, Immunology and Microbiology Section, Immune response, Immunity

## Abstract

Acute Graft-versus-Host Disease (GvHD) remains a major complication of allogeneic haematopoietic stem cell transplantation, with a significant proportion of patients failing to respond to first-line systemic corticosteroids. Reliable biomarkers predicting disease severity and response to treatment are warranted to improve its management. Thus, we sought to determine whether pentraxin 3 (PTX3), an acute-phase protein produced locally at the site of inflammation, could represent a novel acute GvHD biomarker. Using a murine model of the disease, we found increased PTX3 plasma levels after irradiation and at GvHD onset. Similarly, plasma PTX3 was enhanced in 115 pediatric patients on day of transplantation, likely due to conditioning, and at GvHD onset in patients experiencing clinical symptoms of the disease. PTX3 was also found increased in skin and colon biopsies from patients with active disease. Furthermore, PTX3 plasma levels at GvHD onset were predictive of disease outcome since they resulted significantly higher in both severe and therapy-unresponsive patients. Multiple injections of rhPTX3 in the murine model of GvHD did not influence the disease course. Taken together, our results indicate that PTX3 constitutes a biomarker of GvHD severity and therapy response useful to tailor treatment intensity according to early risk-stratification of GvHD patients.

## INTRODUCTION

Acute Graft-*versus*-Host Disease (GvHD) still accounts for 15-30% of allogeneic stem cell transplant (HSCT)-related mortality [1]. Currently, diagnosis of GvHD still relies on the observation of clinical manifestations suggestive of the disease, with histological confirmation, whenever possible. Newly diagnosed patients commonly receive a first-line steroid treatment, with less than 50% of them, however, achieving a sustained response [2]. The outcome of steroid-refractory patients is particularly poor despite the introduction of several second- and third-line therapies [3]. In this scenario, early identification of patients at high risk of developing severe/non responsive GvHD would potentially improve disease management through prompt adoption of risk-tailored therapies.

In recent years, measurement of GvHD-specific biomarkers in the plasma or serum of HSCT patients has emerged as a new diagnostic and prognostic tool [4, 5]. Several cytokines playing a major role in systemic inflammation such as IL-2 and TNF-α have been investigated [5]. Furthermore, soluble molecules associated with allogeneic donor T cell-mediated cytotoxicity on recipient tissues, such as Elafin [6], HGF [7] and REG3α [8], or related to T-cell activation and polarization, such as sTNFRI, sIL-2Rα [7] and ST2 [9, 10], have also been proposed as diagnostic GvHD biomarkers. Among the large number of possible molecules identified, a few have been validated in large cohorts of patients and eventually included in composite biomarker panels [11, 12]. However, the identification of new, highly sensitive, and easily measurable GvHD biomarkers, which could then be validated in a homogeneous allo-HSCT setting, still remains an urgent clinical need. This is particularly true for pediatric patients where the likelihood of developing GvHD, the response to therapy, and non-relapse mortality rates are quite different from adult populations [13].

The long pentraxin 3 (PTX3) is a component of the humoral arm of innate immunity with a recognized non-redundant role in the modulation of inflammation and in the protection against selected pathogens [14]. It is an acute-phase protein rapidly produced by vascular endothelial cells, mesenchymal cells, and fibroblasts, as well as by cells of innate immunity, such as monocytes and granulocytes, upon stimulation by pro-inflammatory cytokines, damaged tissue-derived signals, and microbial molecules [15]. Unlike other acute-phase proteins, such as C-reactive protein, which is almost exclusively produced by hepatocytes in response to inflammation, PTX3 is considered a highly sensitive marker of primary local activation of innate immunity and inflammation, thanks to its peculiar pattern of production. Data obtained in several inflammatory vascular and autoimmune disorders, such as cardiovascular diseases [16], rheumatoid arthritis [17], vasculitis [18], and psoriasis [19], have pointed to a role for PTX3 as inflammatory marker of vascular-bed involvement. In all these conditions, a correlation among PTX3 levels, clinical outcome, and disease activity could be clearly established [20, 21].

In this study, we explored PTX3 as a biomarker of acute GvHD firstly in a murine model of the disease and secondly by prospectively monitoring PTX3 plasma levels in a bi-centric cohort of 115 pediatric patients undergoing HSCT for hemato-oncological diseases. Furthermore, to pursue the design of patient-tailored GvHD therapies, we investigated the predictive power of PTX3 for disease severity and treatment response. Finally, we evaluated the potential role of PTX3 in GvHD pathogenesis taking advantage of a mouse model that recapitulates the human disease.

## RESULTS

### PTX3 plasma levels increase over baseline on day 0 and at disease onset in a mouse model of acute GvHD

**Figure 1:**
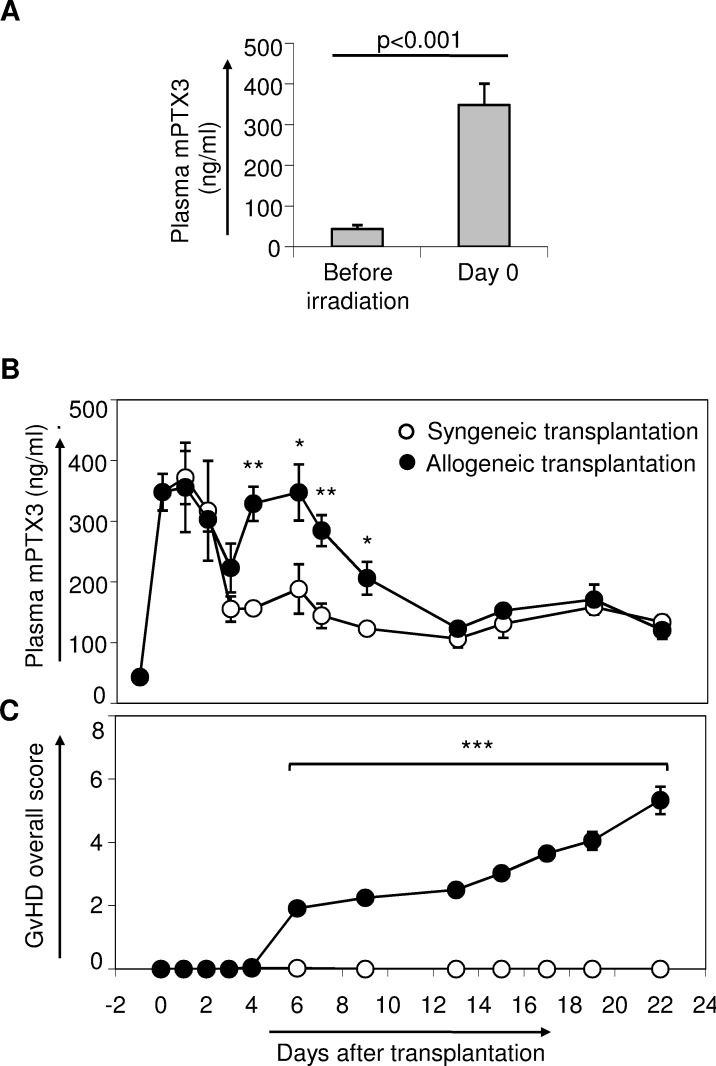
Monitoring of mPTX3 in the plasma of C57Bl/6 mice given either syngeneic or allogeneic transplantation C57Bl/6 mice irradiated with 900 Rad were inoculated intravenously with 10^7^ bone marrow cells and 20 x 10^6^ splenocytes derived from C56Bl/6 donor mice (syngeneic transplantation) or from Balb/c donors (allogeneic transplantation). Data from two independent experiments were pooled (syngeneic controls: *n* = 17; allogeneic transplanted mice: *n* = 18). **A.** mPTX3 plasma levels were evaluated before irradiation (basal, day −1) and on day 0, before transplantation. **B.** Syngeneic and allogeneic transplanted mice were prospectively monitored until day +21 after transplantation in terms of mPTX3 plasma levels (n≥ 5 mice per time point, per group) **C.** and GvHD clinical score. *p≤ 0.05; **p≤ 0.01, ***p≤ 0.001.

To ascertain whether murine PTX3 (mPTX3) might represent a useful biomarker of GvHD severity, we measured PTX3 plasma levels in a murine model of acute GvHD. Interestingly, we observed a significant increase in plasma levels of mPTX3 following irradiation [43.4 ng/ml (SE = 4.17, *n* = 6) at day −1 *vs* 348.21 ng/ml (SE = 30.32, *n* = 6) at day 0, *p* < 0.001] (Figure 1A). Next, we measured mPTX3 plasma levels in either syngeneic or allogeneic transplanted mice until day +21 after transplantation. At day +2, mPTX3 plasma levels started decreasing in both syngeneic and allogeneic grafted mice, reaching a low peak at day +3 (Figure 1B). At day +4, mPTX3 plasma levels in allogeneic, but not syngeneic recipient mice, started rising again reaching a mean value of 329 ng/ml, (SE = 28.1, *n* = 5) at day +4 (Figure 1B). In particular, at day +6, when the first GvHD symptoms became evident (Figure 1C), mean mPTX3 plasma levels in allogeneic recipient mice were significantly higher than those of mice given a syngeneic graft [347.71 ng/ml (SE = 46.29, *n* = 5) *vs* 188.64 ng/ml (SE = 40.73, *n* = 5), *p* = 0.03, respectively]. The protein remained significantly higher in the allogeneic group until day +9, decreasing thereafter to levels comparable to those seen in syngeneic controls.

### PTX3 plasma levels increase after conditioning regimen in a cohort of pediatric patients undergoing allogeneic HSCT

**Figure 2:**
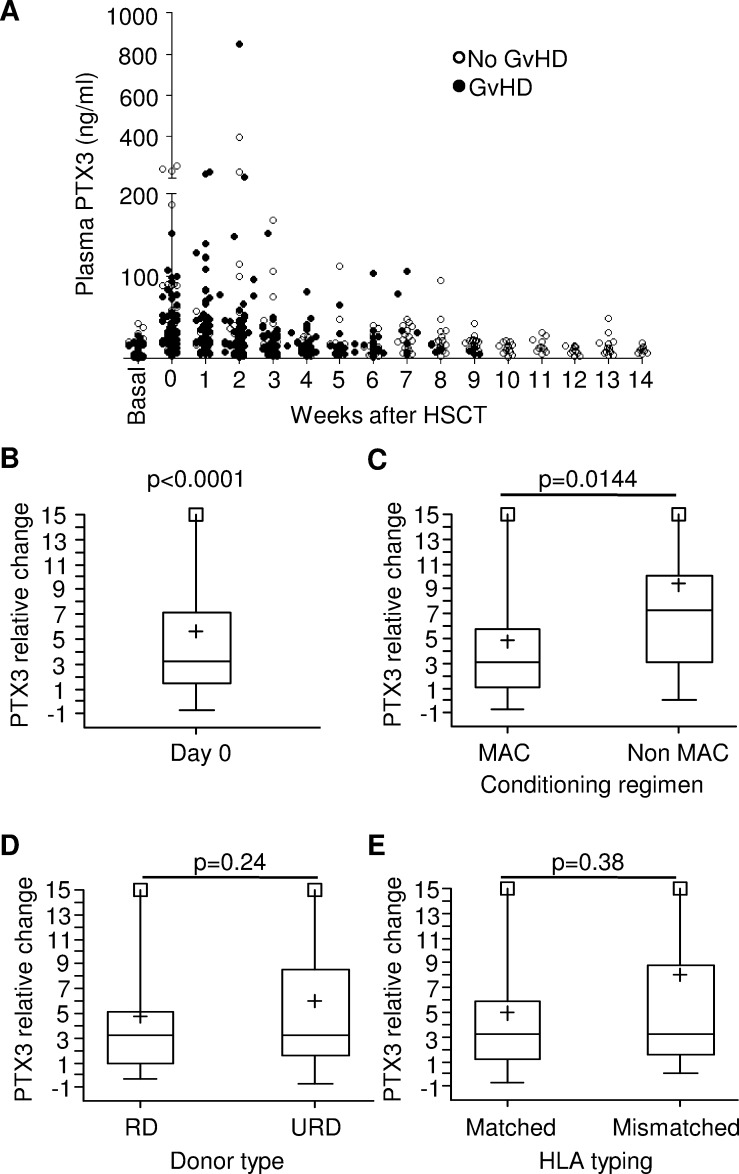
Monitoring and analysis of PTX3 levels in the plasma of HSCT pediatric patients **A.** Available PTX3 plasma levels measured on 115 patients before the beginning of conditioning regimen (basal), on day 0 (HSCT) and weekly until day +100 for patients not experiencing GvHD, or until disease occurrence for GvHD patients. **B.** Relative change (RC) of PTX3 concentration over baseline at day 0 in 96 patients where both measurements could be performed. RC was calculated as follows: day 0 RC = (day 0 PTX3- basal PTX3/basal PTX3). The p-value indicates that RC at HSCT is significantly different from zero. **C.** PTX3 RC at day 0 measured according to the intensity of the conditioning regimen, **D.** the type of donor (RD = related donor, URD = unrelated donor), and **E.** the HLA-matching in the donor/recipient pair (HLA-matched = matching ≥9/10 in the case of bone marrow or peripheral blood; = 6/6 in the case of cord blood). Each box-plot shows the median, the first and third quintiles and extends from the lowest to the highest value; extreme outliers are not shown, but were included in the calculations.

In light of the results obtained in the murine system, we investigated PTX3 plasma levels in a cohort of 115 pediatric patients undergoing HSCT to cure their hemato-oncological diseases. Patients' characteristics, including underlying disease, HSCT type, GvHD prophylaxis and its incidence are summarized in Table I. Figure 2A shows PTX3 plasma levels at the various sampling time points.

**Table 1:** Patients’ characteristics

	GvHD within d100				Overall N=115	
	NO N=38		YES N=77			
**Median age at HSCT, years (range)**	10.2 (2.2, 17.9)		9.3 (0.4, 22.0)		9.5 (0.4, 22.0)	
**Sex, n. *(%)***						
Female	17	*44.7*	20	*26.0*	37	*32.2*
Male	21	*55.3*	57	*74.0*	78	*67.8*
**Disease at HSCT, n. *(%)***						
Acute lymphoblastic leukemia	22	*57.9*	46	*59.7*	68	*59.1*
Acute myeloid leukemia	11	*28.9*	15	*19.5*	26	*22.6*
Chronic myeloid leukemia	1	*2.6*	5	*6.5*	6	*5.2*
Myelodysplastic syndrome	0	*0.0*	5	*6.5*	5	*2.6*
Non-Hodgkin lymphoma	2	*5.3*	2	*2.6*	4	*4.4*
Hodgkin lymphoma	2	*5.3*	1	*1.3*	3	*3.5*
Others	0	*0.0*	3	*3.9*	3	*2.6*
**Graft source, n. *(%)***						
Bone Marrow	28	*73.6*	63	*81.8*	91	*79.1*
Peripheral blood stem cells	5	*13.2*	12	*15.6*	17	*14.8*
Cord blood	5	*13.2*	2	*2.6*	7	*6.1*
**HLA and donor type, n. *(%)***						
HLA ≥9/10, related donor	10	*26.3*	20	*26.0*	30	*26.1*
HLA ≥9/10, unrelated donor	15	*39.5*	43	*55.8*	58	*50.4*
HLA < 9/10, related donor	4	*10.5*	1	*1.3*	5	*4.4*
HLA < 9/10, unrelated donor	4	*10.5*	11	*14.3*	15	*13.0*
HLA 6/6, cord blood	2	*5.3*	1	*1.3*	3	*2.6*
HLA < 6/6, cord blood	3	*7.9*	1	*1.3*	4	*3.5*
**Conditioning, n. *(%)***						
Full intensity (myeloablative)	29	*76.3*	65	*84.4*	94	*81.7*
Reduced intensity	5	*13.2*	6	*7.8*	11	*9.6*
Reduced toxicity	4	*10.5*	6	*7.8*	10	*8.7*
TBI based	17	*44.7*	39	*50.7*	56	*48.7*
Busulfan based	12	*31.6*	25	*32.5*	37	*32.1*
Treosulfan based	4	*10.5*	7	*9.1*	11	*9.6*
Others	5	*13.2*	6	*7.8*	11	*9.6*
**GvHD prophylaxis, n. (*%)***						
Calcineurin inhibitor only	3	*7.9*	3	*3.9*	6	*5.2*
Calcineurin inhibitor + methotrexate	7	*18.4*	11	*14.3*	18	*15.7*
Calcineurin inhibitor + methotrexate + ATG	21	*55.3*	53	*68.8*	74	*64.3*
Other	7	*18.4*	10	*13.0*	17	*14.8*
**GvHD grade at onset, n. *(%)***						
0	38	*100.0*	-	-	38	*33.0*
I	-	-	36	*46.8*	36	*31.3*
II	-	-	37	*48.0*	37	*32.2*
III	-	-	4	*5.2*	4	*3.5*
IV	-	-	0	*0.0*	0	*0.0*

In order to investigate the impact of conditioning regimen on PTX3 plasma levels, we compared PTX3 median values at baseline and at time of HSCT in 96 patients for whom both measurements were available. Interestingly, median PTX3 level significantly increased from a basal value of 7.16 ng/ml (range = 1.00; 42.57) to reach a value of 31.65 ng/ml (range = 2.84; 259.32) at day 0, corresponding to a median relative change (RC) of 3.23 (range = −0.71; 30.40, *p* < 0.0001) (Figure 2B). In addition, we analyzed the impact of known pre-transplant GvHD risk factors, namely conditioning regimen intensity, donor type, and HLA matching, on PTX3 plasma concentration. PTX3 plasma levels at HSCT were significantly higher in 18 patients who underwent reduced toxicity/intensity regimen (median RC = 7.29, range = 0.08; 30.40) as compared to those of 78 patients receiving myeloablative conditioning (MAC) regimen (median RC = 3.08, range = −0.71; 27.65, *p* = 0.014) (Figure 2C). In contrast, no significant differences were observed in PTX3 levels measured at day 0 when we evaluated HSCT recipients according to either donor type or HLA-matching. In detail, patients receiving transplantation from either a related (RD, *n* = 29) or unrelated donor (URD, *n* = 67) had a median RC of 3.22 (range = −0.38; 30.40) and 3.23 (range = −0.71; 27.65) (*p* = 0.24), respectively (Figure 2D). Likewise, median PTX3 RC of 74 patients with a HLA-matched donor was 3.20 (range = −0.71; 24.92) as compared to 3.24 (range = 0.03; 30.40) of 22 patients transplanted from an HLA-disparate donor (*p* = 0.38, Figure 2E).

The increase in PTX3 protein levels was very similar in patients who either did or did not subsequently develop GvHD [median RC = 3.24 (range = −0.>51; 30.40) *vs* 3.20 (range = −0.71; 27.88), *p* = 0.91, respectively], indicating that the increase in PTX3 levels at day 0 was not related to a higher risk of GvHD occurrence within day +100 (Figure S1).

### Plasma PTX3 increases at the onset of GvHD in pediatric patients and is abundantly produced in disease-affected tissues

**Figure 3:**
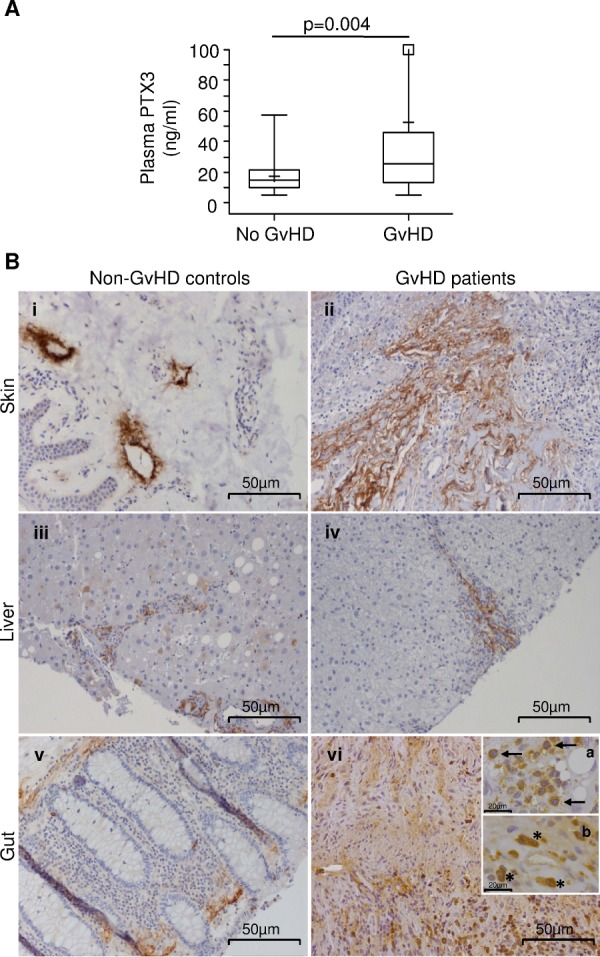
PTX3 levels in the plasma of HSCT patients at disease onset and PTX3 protein production in biopsies from GvHD target organs **A.** PTX3 plasma levels at the onset of acute GvHD (N = 61) and in plasma samples from “no GvHD” patients (N = 38). The box-plot shows the median, the first and third quintiles and extends from the lowest to the highest value; extreme outliers are not shown, but were included in the calculations. **B.** Immunohistochemical analysis of PTX3 expression on paraffin-embedded sections of skin (i, ii), liver (iii, iv) and gut (v, vi) from controls and GvHD patients. Scale bar = 50μm. Insert subpanels vi-a and vi-b show magnified macrophages (↑) and neutrophils (*) infiltrating the gut of a GvHD patient (scale bar = 20μm).

In order to determine the role of PTX3 as potential GvHD biomarker, we analyzed PTX3 levels in children who either did or did not develop the disease within 100 days after HSCT. As shown in Table 1, 77/115 patients (67%) developed acute GvHD within day +100, while 38/115 (33%) did not (“No GvHD” group). The median time to disease onset was 23 days (range = 7; 64 days). PTX3 data were available at GvHD onset for 61/77 (79%) of patients. Noteworthy, the median PTX3 level at the onset of GvHD was 25.30 ng/ml (range = 5.15; 847.44), a value significantly higher than that of the “No GvHD” group [14.49 ng/ml (range = 5.10; 57.44), *p* = 0.004, Figure 3A]. Results did not change if samples collected in the course of any clinically proven infection were excluded (data not shown). Specifically, the median PTX3 level in infection-free “no-GvHD” patients was 12.73 ng/ml (range = 3.80-49.64 ng/ml), significantly lower than that of GvHD patients (*p* = 0.0013).

To further investigate the role of PTX3 in acute GvHD, PTX3 protein production was analyzed by immunohistochemistry on tissues typically affected by the disease, such as skin, liver, and gut, obtained from HSCT patients with active GvHD (Figure 3B). Biopsies of normal tumor-adjacent tissues of cancer patients were used as non-GvHD control. Interestingly, as shown in Figure 3B, a basal perilymphatic expression of PTX3 could be detected in control skin (Panel i), while enhanced PTX3 production in the extracellular matrix was clearly observed in skin affected by GvHD (Panel ii). In contrast, PTX3 production was limited to the portal tract of non-GvHD control and GvHD livers (Panels iii, iv) and to rare hepatocytes in control liver (Panel iii). Furthermore, PTX3 was localized in the subepithelial extracellular matrix in both control and GvHD-involved colon (Panels v, vi). High PTX3 protein levels were further detected in inflammatory cells, such as macrophages (↑) and neutrophils (*) in GvHD-involved colon (Panels vi-a and vi-b).

### Repeated measurements of PTX3 plasma levels over time confirm the diagnostic relevance of PTX3 at GvHD onset, but do not provide overall prediction of GvHD development

Given that weekly measurements of PTX3 were taken from time of HSCT onwards, we also asked whether changes in PTX3 plasma levels at those planned time points could be prognostic of GvHD development. Supplemental Table S1 shows the median plasma PTX3 values observed at various planned time point for patients who did or did not develop GvHD later during follow-up within 100 days. Both subgroups displayed comparable PTX3 plasma levels at day 0. During follow-up, in both subgroups, we observed a gradual decrease in PTX3 plasma levels that never led to significant differences in median PTX3 values between the groups throughout the entire time course.

We further investigated the prognostic role of the joint PTX3 pattern over time using two types of regression models. With the first model, we sought to ascertain whether the risk of GvHD during follow-up could be predicted using all available PTX3 readouts up to the most recent one before GvHD onset (i.e. the one measured in the previous week, as per planned sampling). This regression analysis did not reveal a significant relationship between the pattern of PTX3 plasma levels and the risk of GvHD (*p*-value = 0.71). In contrast, when the model considered all available PTX3 readouts which, for patients who developed GvHD, included the measurement at onset, it revealed that PTX3 had indeed a highly significant diagnostic relevance (*p* < 0.001), suggesting that the protein level rose at time of GvHD occurrence.

### PTX3 plasma levels at GvHD onset are associated with disease severity

**Figure 4:**
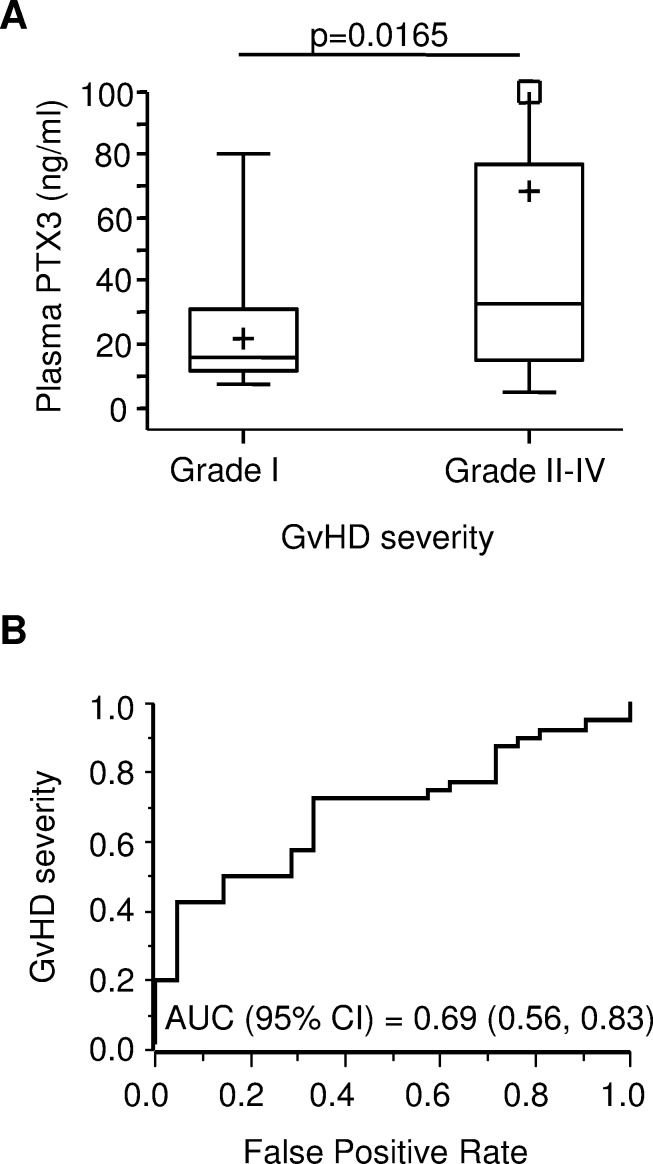
PTX3 plasma levels at GvHD onset by disease severity **A.** PTX3 plasma levels measured at GvHD onset in patients with mild disease (maximum GvHD grade = I, N = 21) and severe disease (maximum GvHD grade II-IV, N = 40). The box-plot shows the median, the first and third quintiles and extends from the lowest to the highest value; extreme outliers are not shown, but were included in the calculations. **B.** Receiver operating characteristic (ROC) curve for the prediction of grade II-IV GvHD severity. AUC = area under the curve; 95% C.I. = 95% confidence interval.

Next, we sought to determine whether PTX3 plasma levels at onset could provide information on GvHD severity. Thus, we analyzed our cohort of patients according to the maximum clinical GvHD grade observed for each patient. PTX3 plasma levels at disease onset in patients with mild GvHD (i.e. maximum grade = I, N = 21) were compared to those of patients with more severe disease (i.e. maximum grade II-IV, N = 40). Remarkably, the median PTX3 level at disease onset was 33.08 ng/ml (range = 5.15; 847.44) in the grade II-IV acute GvHD group, a value significantly higher than that observed in patients who developed mild acute GvHD, whose median PTX3 level was 15.69 (range = 7.27; 80.57, *p* = 0.0165, Figure 4A). The ROC curve for the prediction of grade II-IV GvHD showed an AUC (95% C.I.) of 0.69 [(0.56, 0.83), Figure 4B].

### PTX3 plasma levels at GvHD onset are predictive of poor response to therapy

**Figure 5:**
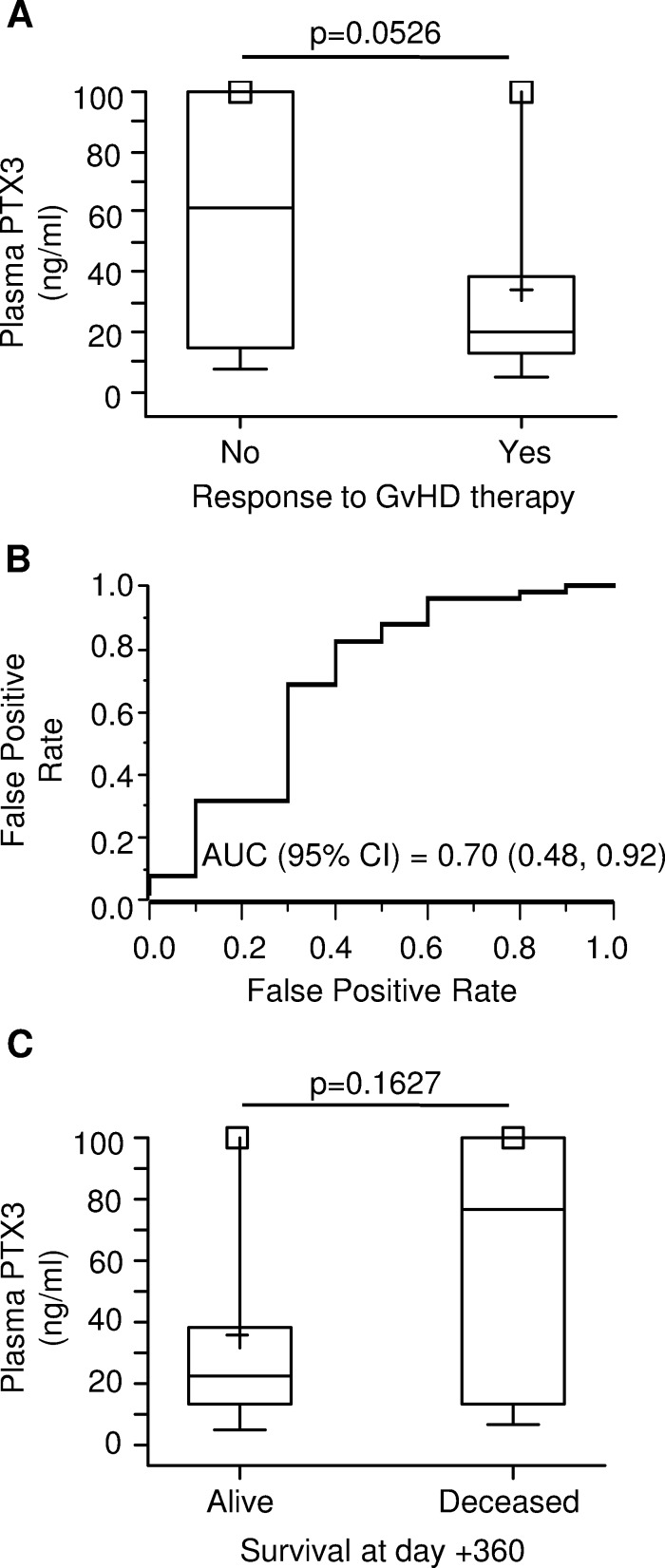
PTX3 plasma levels at GvHD onset by therapy response and survival status at 1-year **A.** PTX3 plasma levels at GvHD onset in patients not responding to anti-GvHD therapy (left, N = 10) and responding completely or partially (right, N = 51). **B.** Receiver operating characteristic (ROC) curve for the prediction of response to anti-GvHD therapy. AUC = area under the curve; 95% C.I. = 95% confidence interval. **C.** PTX3 plasma levels at GvHD onset by status at 1 year after HSCT. The box-plot shows the median, the first and third quintiles and extends from the lowest to the highest value; extreme outliers are not shown, but were included in the calculations.

In an effort to establish a relationship between PTX3 and response to treatment, we measured PTX3 plasma levels in patients who were responsive or not to 4-week GvHD therapy. The median plasma level of PTX3 was 3-fold higher at the time of disease onset in 10 patients who subsequently did not respond to therapy (61.47 ng/ml, range = 7.83; 847.44) than that of 51 patients who displayed either complete or partial response (20.47 ng/ml, range = 5.15; 205.28, *p* = 0.0526) (Figure 5A). The ROC curve for the prediction of response to GvHD treatment showed an AUC (95% C.I.) of 0.70 [(0.48, 0.92), Figure 5B].

Furthermore, we analyzed PTX3 levels at GvHD onset according to the survival status at 1 year after HSCT. Among 77 patients who developed GvHD, 16.9% (*n* = 13) died within the first year after HSCT. PTX3 measurements were available in 9 deceased and 52 surviving patients. As shown in Figure 5C, the median plasma level of PTX3 at GvHD onset was 76.31 ng/ml (range = 7.12; 847.44) in deceased patients. Although not significantly different, this value was more than 3-fold higher than that of surviving patients, whose median PTX3 plasma level was 22.72 ng/ml (range = 5.15; 205.28, *p* = 0.16).

### PTX3 administration does not affect the course of GvHD in allogeneic transplanted mice

**Figure 6:**
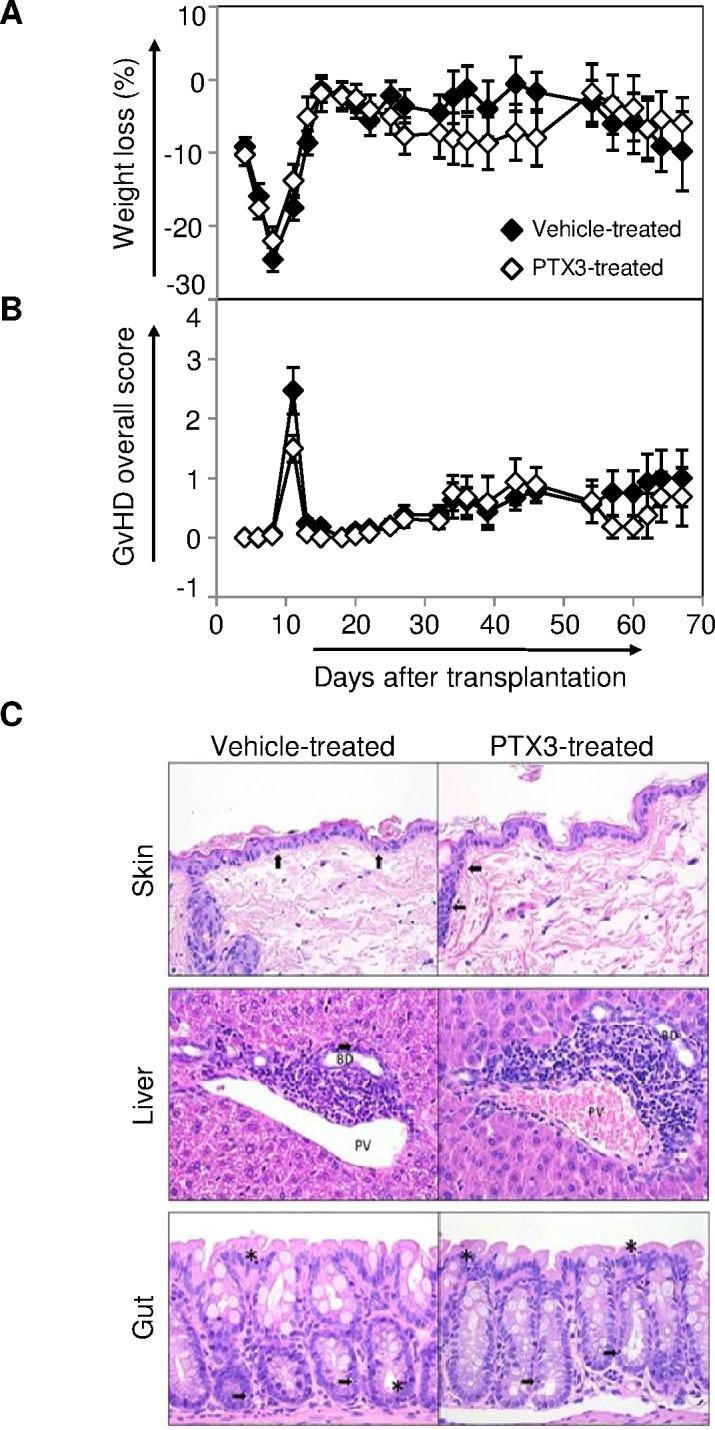
Impact of PTX3 administration on GvHD course in a murine model of the pathology After total body irradiation on day −1, C57Bl/6 recipients were given 7x10^6^ BM cells and 5x10^6^ splenocytes from Balb/c donors in the presence of rhPTX3 three times a week (*n* = 15) or vehicle alone (*n* = 15). All mice, from both groups, survived at least until day +8, and the longest survivors were sacrificed at day +67. **A.** Weight was monitored in transplant recipients until day +67. Data are expressed as mean percentage of weight loss ± standard error. **B.** Mean GvHD overall score ± standard error was calculated for both PTX3 and vehicle treated mice, until day +67. **C.** GvHD histopathological analysis were performed on hematoxylin-eosin stained sections of skin, liver and gut, obtained at day +67 from PTX3 treated mice (*n* = 5) and from vehicle treated mice (*n* = 5), as controls. One representative image per group is shown. Top and middle panels: (↑) intraepithelial lymphocytes, (*) degenerated epithelial cells. Bottom panels: BD = bile duct; PV = portal vein.

We also assessed in the mouse model whether PTX3 could also play a role in disease pathogenesis. For this purpose, we performed repeated injections of recombinant human (rh)PTX3 in our mouse model of GvHD. In order to induce a mild disease, C57Bl/6 recipients were inoculated at day 0 with 7x10^6^ BM cells and 5x10^6^ splenocytes from Balb/c along with i.p. rhPTX3 (*n* = 15) or vehicle alone (*n* = 15) three times per week. As shown in Figure 6A, the extent of weight loss throughout the 67 day-period of observation in rhPTX3-treated and vehicle-treated mice was similar. Likewise, no differences were observed between the two groups in terms of overall GvHD score (Figure 6B).

We next analyzed, at day +67, the appearance of histopathological lesions in skin, liver, and gut from mice treated or not with rhPTX3. As shown in Figure S1, no substantial differences were observed between mice that were injected or not with rhPTX3 in terms of scores attributed to each tissue sample (Figure S2A) as well as total score (Figure S2B). Consistent with the mild GvHD induced by our protocol, we observed moderate lymphocyte infiltration in skin, liver, and colon samples from either group (Figure 6C). Furthermore, we detected similar levels of mild epidermal hyperplasia in the skin of PTX3-treated and vehicle-treated mice (Figure 6C, top panels).

## DISCUSSION

Despite improvement in donor selection and post-transplant immunosuppression, acute GvHD still occurs in 30-90% of patients undergoing allogeneic HSCT and is associated with high mortality rates in those patients experiencing treatment-refractory disease. In particular, transplant-related mortality (TRM) has been estimated to be 46% in patients refractory to corticosteroid therapy and 16% in responders [22, 23]. Several ongoing clinical trials are evaluating the efficacy of other therapies relying on initial GvHD treatment in combination with other treatments originally used as second-line therapy, although the results have so far proved inconclusive ([24, 25] and NCT00609609).

Since PTX3, an acute-phase protein rapidly produced by vascular tissues as well as by cells of innate immunity in response to inflammation [14], is a rapid and reliable marker for primary local activation of innate immunity and inflammation in several autoimmune disorders [17, 19], we sought to determine whether PTX3 levels would change in the plasma of HSCT recipients and whether such changes could be used to predict GvHD severity and patient outcome as early as clinical manifestations appeared. The diagnostic value of such biomarker, in turn, would have allowed us to optimize risk stratification for the design of more effective tailored therapies.

Here, we report that PTX3 is dramatically upregulated in the plasma of syngeneic as well as of allogeneic transplanted mice within the first few hours of conditioning regimen. In good agreement with recent studies on radiation induced-vasculopathy [26], this rapid induction of plasma PTX3 levels could be attributed directly to the inflammatory response of vascular cells along with fibroblasts, epithelial cells, and endothelial cells undergoing irradiation. Interestingly, a second peak of PTX3 induction was only observed in the systemic circulation of allogeneic transplanted mice two days before the appearance of GvHD symptoms. We hypothesize that this increase in PTX3 plasma levels is likely triggered by massive ongoing production of inflammatory cytokines at peripheral sites, which precedes the observation of overt GvHD symptoms, including IL-1, and TNFα [27, 28], which are known to be potent inducers of PTX3 release. Of note, PTX3 plasma concentration started to decrease as the disease progressed. This observation is consistent with published work describing an early elevation of the pro-inflammatory cytokines IL-1α and TNFα in the serum of mice during the first week after transplantation, decreasing over time with concomitant GvHD progression and mortality [29].

Since PTX3 plays a crucial role in inflammation through activation of the complement cascade [30, 31] and inhibition of P-selectin-mediated neutrophil extravasation [32], we sought to establish a relationship between plasma levels of this protein and induction/resolution of GvHD lesions. The timely and continuous administration of PTX3 to allo-transplanted mice did not influence disease course as assessed by the presence of GvHD symptoms and by the histological analysis of disease-affected organs. Thus, PTX3 could represent an early sensor of GvHD-related inflammation, whereas it does not appear to be directly involved in GvHD pathogenesis.

Based on data obtained from the mouse model, we investigated a potential role of PTX3 as a GvHD-related biomarker in a bi-center cohort of 115 pediatric patients undergoing allogeneic HSCT. Consistent with our observations in the murine model, PTX3 plasma levels significantly augmented at day 0 compared with baseline values, most likely due to the conditioning regimen-induced tissue damage. However, this increase did not correlate with known GvHD risk factors, such as the use of unrelated or HLA-disparate donors, nor it appeared to be predictive of a greater risk of developing acute GvHD. Unlike other validated GvHD biomarkers [33], PTX3 levels were higher on day 0 in patients receiving reduced-intensity conditioning (RIC) than in patients given a fully myeloablative regimen. This difference may be due to the persistence of recipient myeloid cells following RIC, which have been shown to produce high amounts of PTX3 in response to inflammatory stimuli [34]. Moreover, RIC patients were homogeneously treated with fludarabine, whose inflammatory effect on monocytes is well established [35].

Interestingly, PTX3 plasma levels, which progressively decreased toward baseline levels in the first few weeks after HSCT, readily increased at the onset of GvHD clinical signs. The high protein concentration in the systemic circulation mirrored PTX3 over-production in GvHD target organs, as demonstrated by immunohistochemical analysis of skin and gastrointestinal lesions. While a basal perivascular and perilymphatic protein expression was detected in skin, liver, and colon biopsies from non-GvHD controls (Doni A., (IRCCS) - Humanitas Clinical and Research Center, Rozzano (MI), Italy, personal oral communication April 2016), PTX3 accumulation was evident in GvHD inflamed dermal and colon extracellular matrix. Under this pathological condition, stromal cells, vascular cells as well as tissue-infiltrating myeloid cells, which are well-known PTX3-producers [15], could have all contributed to the release of PTX3 both in the skin as well as in the gut. Concerning the role of inflammatory effector cells, PTX3-expressing macrophages and neutrophils were detected in GvHD intestinal lesions. PTX3 was not specifically induced in GvHD hepatic lesions, in good agreement with previous data showing that the liver is not a source of PTX3 in inflammatory conditions [30].

PTX3 is an essential component of the innate immune response against several pathogens such as fungi and viruses [36, 37], which often cause severe and even fatal infections in the post-transplant period. Moreover, PTX3 plasma levels are significantly increased in different infectious conditions [38, 39]. Thus, we reasoned that PTX3 plasma level measurements could be somewhat affected by ongoing infections. However, when we compared PTX3 levels at GvHD onset with those measured in patients without GvHD, excluding plasma samples collected from patients suffering from clinically proven infections, we failed to notice significant differences from our previous analysis (data not shown), suggesting robustness of PTX3 as a biomarker for GvHD diagnosis at the onset of clinical symptoms. However, the full pattern of PTX3 readouts, collected at the planned time points up to the one before GvHD onset, could not predict subsequent GvHD occurrence.

Often the GvHD grade at onset is not representative of the ultimate disease severity, which usually becomes evident in 1-2 weeks. It is interesting to note that, in our cohort of patients, PTX3 plasma levels correlated with the maximum overall grade of GvHD, which has been reported to reflect the progression of the disease after treatment [40] and to have a major impact on 2-year disease-free survival [41]. Moreover, patients with treatment-resistant GvHD had higher PTX3 levels at disease onset compared to responders. Likewise, patients who deceased within 1 year after HSCT showed higher protein levels at onset, although the difference was not statistically significant. Our data are in accordance with a recent paper from Doehn and collegues [42] showing that plasma PTX3 levels correlate with the occurrence and severity of acute GvHD in an adult cohort of transplanted patients.

Overall, these results suggest that PTX3 plasma level at GvHD occurrence is a robust onset biomarker and correlates with both maximum disease severity and response to treatment, both these characteristics being potentially useful to timely identify those patients who have a greater probability to die from GvHD, and who therefore could benefit from a more aggressive treatment already at the beginning of the disease [43, 44]. Conversely, our findings suggest that rapid steroid tapering should be considered in children with lower PTX3 plasma levels to avoid over-treating patients that are likely to develop a milder disease. This would also help reduce the risk of developing infectious disease associated with over-treatment. Under current clinical practice, due to the lack of diagnostic tools for risk stratification, all GvHD patients receive the same corticosteroid-based therapy. In recent years, several reports have underscored the clinical need for a biomarker-based and patient-specific GvHD therapy in order to improve disease management [4, 33, 45]. For this purpose, large biomarker panels, believed to provide enough predictive/diagnostic performance, rather than single analytes, are currently under validation in defined cohort of HSCT patients [4, 10, 12].

Here, we have identified PTX3 as an easily measurable disease biomarker able to predict at onset of clinical symptoms, in a pediatric cohort of HSCT patients, the subsequent GvHD severity as well as the likelihood of refractoriness to standard therapy.

Further studies are clearly needed to validate PTX3 as a biomarker that can predict GvHD severity and patient outcome in larger cohorts of HSCT recipients. The inclusion of PTX3 in a composite panel with other validated analytes will constitute a crucial step towards improving GvHD disease management.

## MATERIALS AND METHODS

### Animals

Eight to ten week-old female C57BL/6 (B6, H2b) and Balb/c (H2d) mice were all obtained from Charles River Laboratories (Calco, Milan, Italy). For details, see the Supplemental Methods.

### GvHD mouse model

The GvHD mouse model was performed as previously described [46]. For details, please, see the Supplemental Methods.

### Effect of PTX3 administration on murine GvHD

For details, please, see the Supplemental Methods.

### Patients and samples

One-hundred-fifteen consecutive patients with hemato-oncological diseases, who underwent allogeneic HSCT at the Pediatric Depts. of “San Gerardo Hospital”, Monza and at “Regina Margherita Hospital”, Torino, Italy, were enrolled in the study. Further details are described in the Supplemental Methods.

Peripheral blood samples for PTX3 level measurement were collected before starting the conditioning regimen, on day 0 (i.e. at day of HSCT), weekly after HSCT (day +7, +14, +21, +28 ±3 days etc.) up to day +98 or to GvHD onset, whichever occurred first, and at GvHD onset (before the beginning of GvHD-specific drug therapy). Plasma was obtained after centrifuging whole blood, collected in EDTA containing Vacutainer tubes (Becton Dickinson), at 1000g for 10 minutes. Plasma samples were then frozen at a minimum of −80°C until PTX3 was evaluated by ELISA assay, as previously reported [47].

### Histology

Histological analyses of mouse and human tissues were performed as described in the Supplemental Methods.

### Statistical analysis

#### GvHD mouse model

Student's t test was used to evaluate the differences in terms of GvHD overall score and PTX3 plasma concentration between different groups of mice.

#### PTX3 levels in pediatric recipients of HSCT

The overall impact of HSCT, as well as the impact of each single variable of the procedure (i.e. intensity of conditioning regimen, donor type, and HLA matching), on PTX3 plasma levels was determined by comparing PTX3 levels at HSCT with PTX3 levels before conditioning (baseline). PTX3 relative change (RC) was obtained as follows: RC of PTX3 at HSCT = (PTX3 at HSCT - PTX3 baseline)/PTX3 baseline, and tested by Wilcoxon rank-sum test. In the cohort of patients with GvHD, the impact of PTX3 levels at onset of GvHD on its severity, response to therapy after 4 weeks, and survival status at day +360 after HSCT were assessed by Wilcoxon rank-sum test, as well. The median PTX3 plasma level for “no GvHD” patients was calculated taking into account all available samples and was compared with the median plasma level of PTX3 measured at GvHD onset by Wilcoxon rank-sum test. The impact of PTX3 longitudinal pattern on the risk of GvHD was analyzed by means of a Cox regression model where the level of PTX3 was included as a time-dependent covariate either with or without a lag time of one week. The regression coefficient was tested according to Wald. Logistic regression was applied to investigate the relationship between PTX3 levels at GvHD onset and the probability of developing grade II-IV GvHD and of responding to GvHD treatment. Subsequently, the receiver operating characteristic (ROC) curve was derived. The area under the curve (AUC) was estimated nonparametrically. All tests were two-sided. Analyses were performed with SAS 9.2.

## SUPPLEMENTAL MATERIALS AND METHODS


