# Supplementary material for: Pentraxin 3 plasma levels at graft-versus-host disease onset predict disease severity and response to therapy in children given haematopoietic stem cell transplantation
Source: Oncotarget. 2016 Nov 21;7(50):82123–38. doi: 10.18632/oncotarget.13488 (PMC5347680; doi:10.18632/oncotarget.13488)
Supplement: Supplementary file 1 [file oncotarget-07-82123-s001.pdf]

Pentraxin 3 plasma levels at graft-*versus*-host disease onset predict disease severity and response to therapy in children given haematopoietic stem cell transplantation

Supplemental Materials and Methods

Animals

All animals were housed in dedicated rooms with pathogen-free, individually ventilated cages (IVC, Tecniplast spa, Varese, Italy) in the animal facility of the University of Milano-Bicocca (Monza, Italy) or Humanitas Clinical and Research Hospital. All the procedures involving animals handling and care were in accordance with protocols approved by Milano-Bicocca University and Humanitas Clinical and Research Hospital, in compliance with national and international law and policies. This study was approved by the Italian Ministry of Health (approvals n.9/2011-B, issued on the 01/19/2011, and n.44/2015 PR, issued on 01/28/2015).

Patients' details

One-hundred-fifteen consecutive patients with hemato-oncological diseases were enrolled in the study between March 2009 and November 2013, after that a written informed consent from either parents or legal guardians was obtained. The study was approved by the institutional review boards of the participating Centers. None of the patients included in the study was given a T-cell depleted allograft. At onset of GvHD and at each subsequent time point, the extent of individual organ involvement and the overall grade of acute GvHD were evaluated and recorded, taking into account involvement of all organs with the modified Glucksberg criteria [1, 2]. Prophylaxis and treatment of GvHD were administered according to institutional guidelines or specific clinical transplant protocols, in both cases strictly complying

with international recommendations [3, 4].

Response of GvHD to treatment was evaluated 28 days after onset. Complete response (CR) was defined as the complete resolution of acute GvHD manifestations in all organs. Partial response (PR) was defined as improvement of GvHD grade in at least one of the initially involved organs without complete resolution and without worsening in any other organ. No response (NR) was defined as the same grade of GvHD in all organs or progression of GvHD in any organ.

Conditioning regimens were classified as myeloablative or reduced intensity according to CIBMTR definitions [5, 6] and as reduced toxicity when treosulfan-based [7].

Occurrence of clinically relevant bacterial, fungal, or viral infections during PTX3 monitoring period were recorded for each patient.

GvHD mouse model

As previously described [8, 9], C57BL/6 mice received myeloablative total-body irradiation (900 Rad) using a RADGIL X-Ray treatment unit (Gilardoni, Mandello del Lario, Italy) in two fractioned doses, with a 2-h interval. Within 24 hrs from irradiation, C57BL/6 mice were i.v. infused with $10x10^6$ BM cells and $20x10^6$ splenocytes from C57BL/6 (syngeneic transplantation, n=17) or from Balb/c donors (allogeneic transplantation, n=18) and were given water containing gentamycin (80 mg/l) starting one week before irradiation and for all the length of the experiment. Transplanted mice were monitored for GvHD symptoms until day +22. All mice remained alive in the monitored period. GvHD severity was assessed using a previously described scoring system [10] based on five parameters indicative of disease symptoms: skin

integrity, fur texture, activity, posture, and diarrhea, using a score of 0 to 2, with 0 for absent or normal, 1 for mildly abnormal, and 2 for severely abnormal. The GvHD overall score was the sum of the scores for each single criterion. Blood samples were collected from mice tail veins (at least 5 animals for each time point), from day -1 (before irradiation) up to day +22 after transplantation, into EDTA containing Microtainer® tubes (Becton Dickinson, Milan, Italy). Blood samples were centrifuged at 1000g for 10 minutes to obtain plasma, which was then cryopreserved at -80°C until measurement of murine PTX3 levels by ELISA (R&D Systems, Minneapolis, MN).

Effect of PTX3 administration on murine GvHD

To take advantage of a model of mild acute GvHD, $7x10^6$ BM cells and $5x10^6$ splenocytes obtained from Balb/c mice were transplanted in C57BL/6 recipients, after 900 Rad irradiation. Due to the high sequence homology between human and murine PTX3 (more than 80% identical nucleotides in their cDNA sequences) [11], mice were treated three times per week i.p. with 1 mg/Kg of recombinant human (rh)PTX3 (Sigma Tau, Pomezia, Italy) diluted in saline. Control mice were treated with saline alone (vehicle-treated). Intestinal tract, liver, and dorsal skin were sampled from mice at day +67 (5 mice per group).

Hystology

Histopathological analysis of mouse samples was performed on tissues fixed in 10% neutral buffered formalin (NBF). Paraffin-embedded 4-µm sections from each sample were stained with hematoxylin-eosin (HE) and evaluated under a light microscope (Leica DM 2500). GvHD lesions of skin, liver, and gut were scored according to the

histological GvHD grading scale provided by Fowler et al. [12], slightly modified to adapt it to the findings of this study. Scores of individual organs (range 0-4) were added to provide a total GvHD score for each mouse (range 0-12). Human formalin-fixed paraffin embedded tissues were cut at 3 µm. After dewaxing and rehydration, antigen unmasking was performed in 250mM EDTA buffer pH 8.00 in a microwave oven for 10 min at 800W. Endogenous peroxidase was blocked with 2% H_2O_2 for 20 min and unspecific binding sites were blocked with 2% BSA for 30 min. Affinity-purified rabbit IgG against human PTX3 was diluted to 250 ng/ml and incubated for 1h at room temperature in humid chamber. The reaction was revealed by the biotin-free peroxidase detection system (MACH 1 Universal HRP Polymer; BioCare Medical, USA), with 3,3'-diaminobenzidine (DAB; BioCare Medical) free base as chromogen. Normal tumor-adjacent tissue from cancer patient biopsies was used as non-GvHD control.

Supplemental References

1. Filipovich AH, Weisdorf D, Pavletic S, Socie G, Wingard JR, Lee SJ, Martin P, Chien J, Przepiorka D, Couriel D, Cowen EW, Dinndorf P, Farrell A, et al. National Institutes of Health consensus development project on criteria for clinical trials in chronic graft-versus-host disease: I. Diagnosis and staging working group report. Biol Blood Marrow Transplant. 2005; 11: 945-56. doi: 10.1016/j.bbmt.2005.09.004.

2. Przepiorka D, Weisdorf D, Martin P, Klingemann HG, Beatty P, Hows J, Thomas ED. 1994 Consensus Conference on Acute GVHD Grading. Bone Marrow Transplant. 1995; 15: 825-8. doi:

3. Ruutu T, Gratwohl A, de Witte T, Afanasyev B, Apperley J, Bacigalupo A, Dazzi F, Dreger P, Duarte R, Finke J, Garderet L, Greinix H, Holler E, et al. Prophylaxis and treatment of GVHD: EBMT-ELN working group recommendations for a standardized practice. Bone Marrow Transplant. 2014; 49: 168-73. doi: 10.1038/bmt.2013.107.

4. Dignan FL, Clark A, Amrolia P, Cornish J, Jackson G, Mahendra P, Scarisbrick JJ, Taylor PC, Hadzic N, Shaw BE, Potter MN, Haemato-oncology Task Force of British Committee for Standards in H, British Society for B, et al. Diagnosis and management of acute graft-versus-host disease. Br J Haematol. 2012; 158: 30-45. doi: 10.1111/j.1365-2141.2012.09129.x.

5. Giralt S, Ballen K, Rizzo D, Bacigalupo A, Horowitz M, Pasquini M, Sandmaier B. Reduced-intensity conditioning regimen workshop: defining the dose spectrum. Report of a workshop convened by the center for international blood and marrow transplant research. Biol Blood Marrow Transplant. 2009; 15: 367-9. doi: 10.1016/j.bbmt.2008.12.497.

6. Gyurkocza B, Sandmaier BM. Conditioning regimens for hematopoietic cell transplantation: one size does not fit all. Blood. 2014; 124: 344-53. doi: 10.1182/blood-2014-02-514778.

7. Danylesko I, Shimoni A, Nagler A. Treosulfan-based conditioning before hematopoietic SCT: more than a BU look-alike. Bone Marrow Transplant. 2012; 47: 5-14. doi: 10.1038/bmt.2011.88.

8. Calcaterra C, Sfondrini L, Rossini A, Sommariva M, Rumio C, Menard S, Balsari A. Critical role of TLR9 in acute graft-versus-host disease. J Immunol. 2008; 181: 6132-9. doi:

9. Zanotti L, Sarukhan A, Dander E, Castor M, Cibella J, Soldani C, Trovato AE, Ploia C, Luca G, Calvitti M, Mancuso F, Arato I, Golemac M, et al. Encapsulated mesenchymal stem cells for in vivo immunomodulation. Leukemia. 2013; 27: 500-3. doi: 10.1038/leu.2012.202.

10. Cooke KR, Kobzik L, Martin TR, Brewer J, Delmonte J, Jr., Crawford JM, Ferrara JL. An experimental model of idiopathic pneumonia syndrome after bone marrow transplantation: I. The roles of minor H antigens and endotoxin. Blood. 1996; 88: 3230-9. doi:

11. Introna M, Alles VV, Castellano M, Picardi G, De Gioia L, Bottazzai B, Peri G, Breviario F, Salmona M, De Gregorio L, Dragani TA, Srinivasan N, Blundell TL, et al. Cloning of mouse ptx3, a new member of the pentraxin gene family expressed at extrahepatic sites. Blood. 1996; 87: 1862-72. doi:

12. Fowler DH, Kurasawa K, Smith R, Eckhaus MA, Gress RE. Donor CD4-enriched cells of Th2 cytokine phenotype regulate graft-versus-host disease without impairing allogeneic engraftment in sublethally irradiated mice. Blood. 1994; 84: 3540-9. doi:

Supplemental Table S1

Median (range) plasma level of PTX3 at various time points and subsequent GvHD occurrence

TIME POINT		GvHD within d100		p-value
		NO	YES	
DAY 0	N	36	71	
	PTX3 level median (range)	32.15 (6.24; 259.32)	31.29 (2.84; 151.64)	0.3462
DAY 7	N	33	64	
	PTX3 level median (range)	21.78 (5.14; 122.31)	23.96 (5.25; 227.28)	0.3048
DAY 14	N	34	52	
	PTX3 level median (range)	17.70 (1.83; 395.20)	15.23 (3.26; 65.62)	0.3940
DAY 21	N	26	34	
	PTX3 level median (range)	14.85 (3.80; 168.15)	12.93 (2.40; 50.31)	0.3181
DAY 28	N	23	17	
	PTX3 level median (range)	10.67 (2.74; 34.94)	10.41 (3.51; 49.13)	0.6640
DAY 35	N	20	13	
	PTX3 level median (range)	10.25 (4.42; 111.89)	10.57 (5.51; 65.02)	0.8407

Time points are relative to the planned PTX3 sampling days from HSCT (day 0) up to day +35. Subsequent time points are not reported due to small numbers. At each time point, patients who had already developed GvHD were excluded. N= number of patients with available material for PTX3 measurement at each time point.

Supplemental Figures

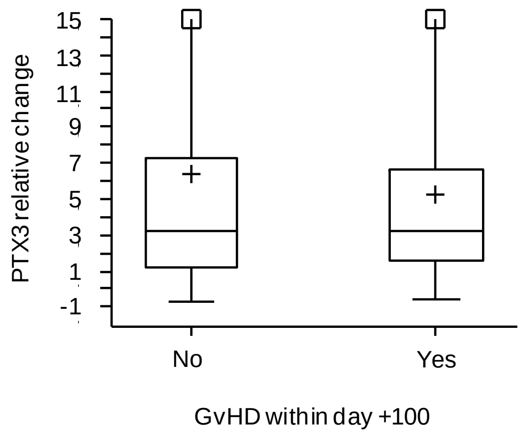

Figure S1: Analysis of PTX3 levels in the plasma of patients developing or not GvHD within 100 days from HSCT. The relative change (RC) of PTX3 concentration over baseline at day 0 was calculated in 96 patients where both measurements could be performed as follows: day 0 RC=(day 0 PTX3- basal PTX3/ basal PTX3). PTX3 RC at day 0 was compared between patient groups developing or not GvHD within 100 days from HSCT. Each box-plot shows the median, the first and third quintiles and extends from the lowest to the highest value; extreme outliers are not shown, but were included in the calculations.

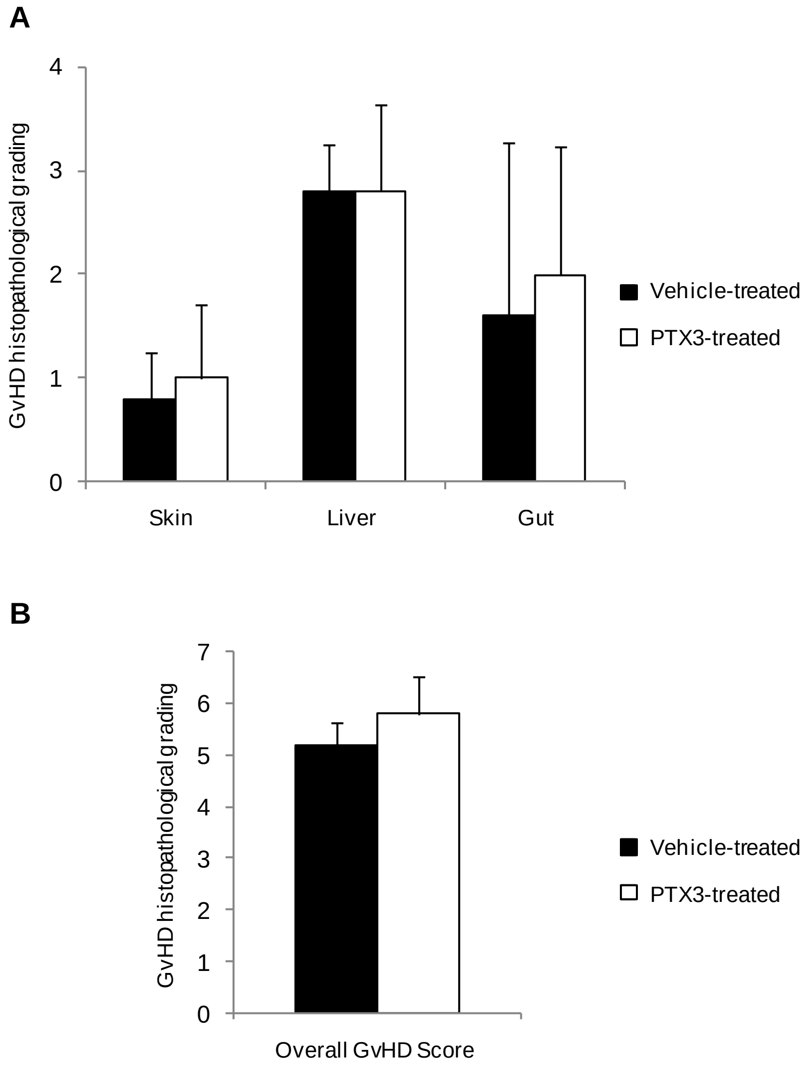

Figure S2: Histopathological grading of GvHD mice receiving or not recombinant human PTX3. (A) Mean single organ score ± standard deviation and **(B)** mean overall GvHD score ± standard deviation were evaluated in allogeneic transplanted C57Bl/6 mice treated three times per week with recombinant human PTX3 (n=5) or vehicle alone (n=5).
